# Weak expression of cyclooxygenase-2 is associated with poorer outcome in endemic nasopharyngeal carcinoma: analysis of data from randomized trial between radiation alone versus concurrent chemo-radiation (SQNP-01)

**DOI:** 10.1186/1748-717X-4-23

**Published:** 2009-07-10

**Authors:** Susan Li Er Loong, Jacqueline Siok Gek Hwang, Hui Hua Li, Joseph Tien Seng Wee, Swee Peng Yap, Melvin Lee Kiang Chua, Kam Weng Fong, Terence Wee Kiat Tan

**Affiliations:** 1 Department of Radiation Oncology, National Cancer Centre, Singapore; 2 Department of Pathology, Singapore General Hospital, Singapore; 3 Divison of Cellular and Molecular Research, National Cancer Centre, Singapore; 4 Division of Clinical Trials and Epidemiological Sciences, National Cancer Centre, Singapore

## Abstract

**Background:**

Over-expression of cyclooxygenase-2 (COX-2) enzyme has been reported in nasopharyngeal carcinoma (NPC). However, the prognostic significance of this has yet to be conclusively determined. Thus, from our randomized trial of radiation versus concurrent chemoradiation in endemic NPC, we analyzed a cohort of tumour samples collected from participants from one referral hospital.

**Methods:**

58 out of 88 patients from this institution had samples available for analysis. COX-2 expression levels were stratified by immunohistochemistry, into negligible, weak, moderate and strong, and correlated with overall and disease specific survivals.

**Results:**

58% had negligible or weak COX-2 expression, while 14% and 28% had moderate and strong expression respectively. Weak COX-2 expression conferred a poorer median overall survival, 1.3 years for weak versus 6.3 years for negligible, 7.8 years, strong and not reached for moderate. There was a similar trend for disease specific survival.

**Conclusion:**

Contrary to literature published on other malignancies, our findings seemed to indicate that over-expression of COX-2 confer a better prognosis in patients with endemic NPC. Larger studies are required to conclusively determine the significance of COX-2 expression in these patients.

## Introduction

Nasopharyngeal carcinoma (NPC) is the sixth most common male cancer in Singapore. The current standard of care for locally advanced NPC is concurrent chemo-radiation, which is associated with increased acute and long term morbidities [1,2]. Increasing effort has been directed toward developing molecular targeted therapies for the treatment of NPC with increasing interest in cyclooxygenase-2 (COX-2) inhibitors.

COX-2 is a 68 kDA enzyme that catalyses the conversion of arachidonic acid to prostaglandins. Over-expression of COX-2 has been found in a variety of malignancies, both gastrointestinal (colon, oesophagus, stomach, pancreas) as well as outside the gastrointestinal tract (lung, breast, bladder and cervix), and shown to correlate with poorer outcomes [3-6].

We hereby describe a retrospective analysis of 58 samples from patients, diagnosed with endemic NPC, who had previously been randomized into a trial of radiotherapy (RT) alone versus concurrent chemo-radiation (CRT) [7]. The aims of the study were to determine the expression level of COX-2 in our cohort of patients and to correlate this with known prognostic factors and overall and disease free survival. We thought the latter would be of particular interest given that studies pertaining to the prognostic significance of COX-2 expression in endemic NPC have so far delivered mixed results [8,9].

## Materials and methods

### Patients

Between September 1997 to May 2003, 221 patients were accrued into a randomized phase III trial (SQNP01) comparing RT alone to CRT in patients with World Health Organization type II or III NPC [7]. All patients had stage III or IVA/B NPC [10]. Patients on the RT alone arm received standard-course RT to a dose of 70 Gy in 35 fractions using a modified Ho's technique. Patients on the CRT arm received 3 cycles of concurrent cisplatin on weeks 1, 4 and 7 of RT, followed by a further 3 cycles of adjuvant 5-fluorouracil and cisplatin.

Of the 221 patients, 88 were referred for treatment from a single institution following initial diagnosis of NPC. For logistic reasons, only patients from this hospital were included in this study. 58 out of these 88 patients had sufficient pre-treatment paraffin-embedded biopsy material available for analysis.

Institutional review board approval was obtained.

### Immunohistochemistry

Archived paraffin blocks of tumor tissue biopsies were sectioned at 4 μm, dewaxed and rehydrated in a graded series of alcohol. This was followed by blockage of endogenous peroxidase in 3% hydrogen peroxide (H2O2) and 0.1% protease, digested for 2 minutes at room temperature. The sections were incubated with COX-2 mouse monoclonal antibody (Neomarkers RM9121-S, Clone SP21, Thermo Fisher Scientific, Cheshire, UK) diluted 1:500 overnight at room temperature. The slides were then washed in 3 changes of tris-buffered saline (pH 7.6) for 2 minutes each before incubation with Dako Envision+ System, Peroxidase (Dako, Glostrup, Denmark) for 30 minutes at room temperature. The peroxidase-catalyzed product was then visualized using Biogenex DAB Chromogen Kit (Biogenex, San Ramon, CA). The specimen was counterstained with Harris Haematoxylin, dehydrated, cleared and mounted in dibutyl-phthalate xylene (DPX) for analysis.

### Quantitation

A semi-quantitive immunohistochemical (IHC) assay was used to determine the level of COX-2 expression. A single head and neck histopathologist was assigned to perform the scoring. She was blinded to all patient characteristics including the treatment received. The extent of COX-2 staining was scored from 0 to 3, and the intensity of staining scored from 1 to 4. The scores were then multiplied together and the final scores classified as follow: 0, negligible staining; 1–4, weak staining; 5–8, moderate staining; and 9–12, strong staining. For the purpose of statistical analysis, the cohort was grouped into tumors with negligible or weak staining (N = 34) versus tumors with moderate or strong staining (N = 24) as well as according to the 4 expression levels above.

### Statistical analysis

Student's t-test was used to compare the age between patients with COX-2 IHC and those without COX-2 IHC. Similarly, Fisher's exact test was performed to compare the sex, T status, N status, TNM stage and treatment received between these two groups of patients. Among patients with COX-2 IHC, Fisher's exact test was used to investigate the distribution of IHC scores among those with different N stage, T stage, TNM stage and treatment received. Overall survival and disease specific survival (DSS) (defined as the period from the date of randomization to the date of death due to the disease or the date of the last follow up, whichever is earlier) was analyzed using Kaplan-Meier method and compared using log-rank test. Hazards ratio (HR), together with 95% confidence interval (CI), was reported by means of Cox regression.

## Results

### Patient characteristics

Archival material for IHC analysis was available for 58 out of 88 patients referred from one institution and enrolled into SQNP-01. The total number of patients randomized into this trial was 221. The median follow-up duration was 4.95 years. The characteristics of these 58 patients are summarized in Table 1. Compared with the group of patients without COX-2 analysis data (the balance of the 221 patients), there was no significant difference in age, gender distribution, T status, N status, TMN stage and treatment allocated between the groups.

**Table 1:** Patients' characteristics

Characteristic	Patients with COX-2 IHC No. (%) (N = 58)	Patients without COX-2 IHC No. (%) (N = 163)	*P*
Age (years)			

Median (Range)	44 (30–74)	46 (14–76)	0.594

			

Sex			

Male	49 (84.5%)	131 (80.4%)	

Female	9 (15.5%)	32 (19.6%)	0.559

			

T status			

1	9 (15.5%)	19 (11.7%)	

2	13 (22.4%)	52 (31.9%)	

3	17 (29.3%)	48 (29.5%)	

4	19 (32.8%)	44 (27.0%)	0.497

			

N status			

0	4 (6.9%)	19 (11.7%)	

1	10 (17.2%)	18 (11.0%)	

2	23 (39.7%)	85 (52.2%)	

3	21 (36.2%)	41 (25.2%)	0.143

			

TNM stage			

II	-	1 (0.6%)	

III	25 (43.1%)	80 (49.1%)	

IV	33 (56.9%)	82 (50.3%)	0.592

			

Treatment			

RT	27 (46.6%)	83 (50.9%)	

CRT	31 (53.4%)	80 (49.1%)	0.647

Abbreviations: COX-2, cyclooxygenase-2; IHC, immunohistochemistry; RT, radiation; CRT, chemo-radiation.

### COX-2 expression, its correlation with known prognostic factors and its impact on overall and disease specific survivals

**Figure 1:**
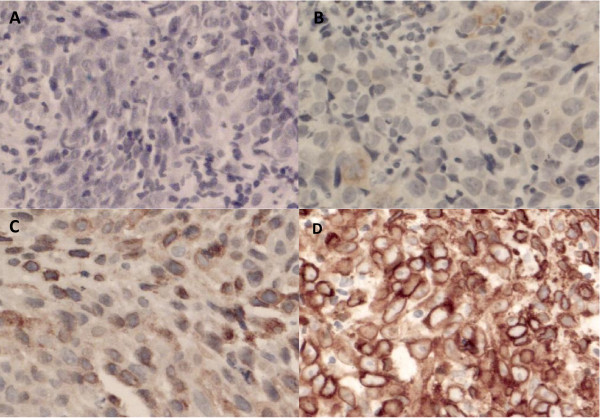
**COX-2 Immunohistochemistry Patterns – typical examples**. A: Negative staining for COX-2 ×200. B: Weak staining for COX-2 ×200. C: Moderate staining for COX-2 ×200. D: Strong staining for COX-2 ×200.

Among the samples analyzed, 58% demonstrated negligible or weak COX-2 expression (29%, negligible; 29%, weak) and 42% showed moderate or strong expression (14%, moderate; 28%, strong), typical examples are shown in figure 1.

**Figure 2:**
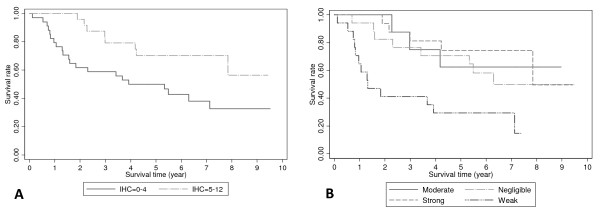
**Kaplan-Meier survival curves according to immunohistochemistry (IHC) scores for negligible and weak cyclooxygenase-2 (COX-2) expression (IHC scores 0–4) versus moderate and strong COX-2 expression (IHC scores 5–12) (p = 0.023; A), and the individual stratified categories (p = 0.002; B), analyzed using the log-rank test**.

**Figure 3:**
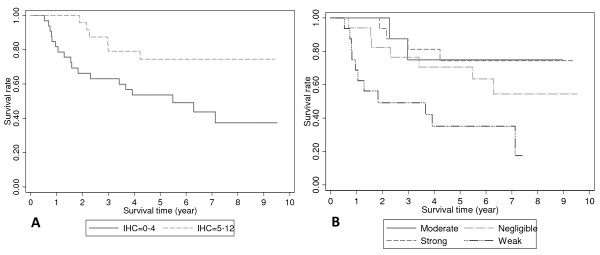
**Kaplan-Meier disease specific survival according to IHC scores for negligible and weak cyclooxygenase-2 (COX-2) expression (IHC scores 0–4) versus moderate and strong COX-2 expression (IHC scores 5–12) (p = 0.020; A), and the individual stratified categories (p = 0.006; B), analyzed using the log-rank test**.

Univariate analysis showed that overall survival was significantly better for patients with tumors demonstrating moderate or strong COX-2 expression (IHC score 5–12) than those whose tumors showed negligible or weak expression (IHC score 0–4) (Figure 2A; p = 0.023). The median overall survival for patients with tumours with negligible or weak COX-2 expression was 5.3 years, while it was not achieved for patients with tumours with moderate or strong COX-2 expression: patients in this group having a lower risk of death with a hazard ratio of 0.40 (95% CI: 0.18 to 0.90). When analyzed according to the stratified expression levels, patients with weak COX-2 expression were found to have the worst median survival (1.3 years versus 6.3 years for negligible; and 7.8 years for strong; median survival was not reached for patients with moderate COX-2 expression; Figure 2B; p = 0.002). Disease specific survival followed the same pattern, the median DSS for patients whose tumors had negligible or weak COX-2 expression was 5.49 years while it was not reached for patients whose tumors had moderate or strong expression (p = 0.020, Figure 3A). Again, when analysed according to the stratified expression levels, patients whose tumors had weak COX-2 expression had a median DSS of 1.83 years, while the median DSS for the other 3 groups was not reached (p = 0.006, Figure 3B).

Multivariate analysis was not performed due to small patient numbers. Instead we examined for correlation between COX-2 expression and the following prognostic factors: treatment received (RT versus CRT) (Table 2), T status, N status and TMN stage. The only correlation which reached statistical significance was between T status and COX-2 IHC scores (p = 0.029); it was observed that within the T1 tumors, there was none which showed moderate or strong COX-2 expression (Table 3). Also, comparing the overall survival for patients with N<3 versus N3 among those demonstrating weak COX-2 expression, there was no difference in overall survival between the groups (1.8 years versus 1.3 years; p = 0.747). Local recurrence and other patterns of failure were not analyzed as there were too few events.

**Table 2:** Distribution of patients with different IHC score by treatment

IHC score	CRT	RT
Negligible (0)	8	9

Weak (1–4)	10	7

Moderate (5–8)	4	4

Strong (9–12)	9	7

Abbreviations: IHC, immunohistochemistry; RT, radiation; CRT, chemoradiation.

Fisher's exact test showed no significant difference of IHC expression levels between patients who received RT versus CRT levels (p = 0.933).

**Table 3:** Distribution of T and N status by IHC scores

	IHC score			
T status	0	1–4	5–8	9–12

1	6	3	0	0

2	3	3	2	5

3	4	3	1	9

4	4	8	5	2
				

				

N status				

0	1	0	2	1

1	3	2	1	4

2	8	5	2	8

3	5	10	3	3

Abbreviations: IHC, immunohistochemistry. Fisher's exact test showed that the distribution of IHC score among patients with different T status was significantly different (p = 0.019), while there was no difference among patients with different N status (p = 0.295).

## Discussion

To the best of our knowledge, this is only the second study examining COX-2 expression in NPC that involved a cohort of patients treated uniformly as part of a clinical trial. Although the 58 patients were only a subgroup of the entire randomized cohort, they were nonetheless representative as there was no difference in patient, tumor or treatment characteristics between them and the remainder of patients where no COX-2 analysis was performed.

Based on our findings, 71% of NPC expressed COX-2, in keeping with other series which reported similar proportions in the range of 62% to 83% [8,9,11,12]. In the only other report based on a cohort of patients receiving treatment as part of a randomized trial, Chan et al. [8] reported that the proportion of biopsies showing negligible, weak, moderate or strong intensity of COX-2 staining was 17%, 24%, 32% and 27% respectively; whereas in our study the corresponding figures were 29%, 29%, 14% and 28%. In that same study, univariate analysis showed that patients whose tumors co-expressed COX-2 and hypoxia-inducible factor-1alpha (HIF-1alpha) experienced inferior progression free survival, though this finding was not significant on multivariate analysis. This is in contrast to our findings where patients with weak COX-2 expression were associated with inferior overall and disease specific survival. Unfortunately, our small sample size precluded multivariate analysis, but test for correlation found a statistically significant imbalance in the T status, with T1 tumors having only negligible or weak COX-2 expression. This finding could have suggested that perhaps, insignificant COX-2 expression may be associated with a smaller primary tumour, hence theoretically resulting in better outcomes. However, our results indicated otherwise.

There had been numerous studies analyzing COX-2 expression and their prognostic significance across a multitude of malignancies. They included studies with large sample sizes treated with uniform protocols in a randomized clinical trial setting [13,14]. In those studies, overexpression of COX-2 was reported to confer a worse prognosis, specifically in those with certain tumour characteristics and having underwent specific treatment modality. These were chiefly prostate tumors treated by radiotherapy and short-term hormonal treatment, breast cancers which were estrogen-receptor positive and treated by breast conserving surgery and radiotherapy, and rectal cancers which received preoperative radiotherapy. To allow us to more appropriately apply COX-2 as a prognostic marker, a better understanding of the mechanisms of interaction between COX-2 and the individual treatment modalities is much needed.

The possible mechanisms that underlie the association of weak, rather than COX-2 overexpression with worse overall survival in endemic NPC is outside the scope of our present study. A plausible explanation could be suggested by a separate finding described in hepatocellular carcinoma, also a viral-associated cancer endemic in our population, where COX-2 was found to be overexpressed in the well-differentiated sub-types and was correlated with the presence of pro-inflammatory cells, macrophages and mast cells [15,16]. Alternatively, COX-2 overexpression had been shown to confer a growth disadvantage by inducing cell cycle arrest via a prostaglandin-independent mechanism [17].

Given the discrepancy between the 2 studies in endemic NPC for which analysis was performed on defined patient cohorts treated uniformly in a clinical trial (albeit the study by Chan et al. [8] included stage II patients while our study only involved stage III and IV patients), future studies with a larger sample size (assuming the same proportion of COX-2 expression in NPC as reported in our series, approximately 160 patients will be required for multivariate analysis) should be performed to show conclusively if weak COX-2 expression independently confers a worse prognosis in these patients and if so, elucidate the mechanisms involved.

## Competing interests

The authors declare that they have no competing interests.

## Authors' contributions

SL, YSP, FKW, JW and TT conceived of the study, participated in its design and coordination. SL, TT and MC drafted the manuscript. JH performed the immunohistochemistry analysis. LHH performed the statistical analysis and contributed the figures.
